# Bioengineering Approaches to Mature Human Pluripotent Stem Cell-Derived Cardiomyocytes

**DOI:** 10.3389/fcell.2017.00019

**Published:** 2017-03-09

**Authors:** Xuetao Sun, Sara S. Nunes

**Affiliations:** ^1^Toronto General Research Institute, University Health NetworkToronto, ON, Canada; ^2^Institute of Biomaterials and Biomedical Engineering, University of TorontoToronto, ON, Canada; ^3^Heart & Stroke/Richard Lewar Centre of Excellence, University of TorontoToronto, ON, Canada

**Keywords:** cardiomyocytes, cardiac regeneration, stem cell, biomaterials, cell therapy, electrical stimulation, mechanical stimulation

## Abstract

Human pluripotent stem cell-derived cardiomyocytes (hPSC-CM) represent a potential unlimited cell supply for cardiac tissue engineering and possibly regenerative medicine applications. However, hPSC-CMs produced by current protocols are not representative of native adult human cardiomyocytes as they display immature gene expression profile, structure and function. In order to improve hPSC-CM maturity and function, various approaches have been developed, including genetic manipulations to induce gene expression, delivery of biochemical factors, such as triiodothyronine and alpha-adrenergic agonist phenylephrine, induction of cell alignment in 3D tissues, mechanical stress as a mimic of cardiac load and electrical stimulation/pacing or a combination of these. In this mini review, we discuss biomimetic strategies for the maturation for hPSC-CMs with a particular focus on electromechanical conditioning methods.

## Introduction

Human embryonic stem cells (hESCs), first isolated from inner cell mass of blastocysts, possess the capacity to differentiate into cells of all three germ layers (Thomson et al., 1998). Similar characteristics can also be found in human induced pluripotent stem cells (hiPSCs), which are generated from terminally differentiated, adult cells by genetically reprogramming via expression of a set of transcription factors (Takahashi et al., 2007; Yu et al., 2007). These cells circumvent the ethical concerns associated with hESCs and allow a potential autologous approach without the need for long-term immunosuppression. Cardiomyocytes can be differentiated from both hESCs and hiPSCs using directed differentiation approaches, which are based on the stage-specific treatment with cardiogenic-inducing signaling factors (Laflamme et al., 2007; Yang et al., 2008).

However, human pluripotent stem cell derived cardiomyocytes (hPSC-CMs) (including hESC-CM and hiPSC-CM) display immature characteristics when compared to adult cardiomyocytes, such as (Table 1):

1. Genetically, hPSC-CMs express much lower levels of cardiac contractile and cytoskeletal genes (Cao et al., 2008; Xu et al., 2009). Early hPSC-CMs have high proliferation rates (Robertson et al., 2013) while adult cardiomyocytes are considered non-proliferative (~0.5% proliferation per year) (Bergmann et al., 2009).
2. Morphologically, hPSC-CMs are small, disorganized, mononucleated, round/triangular in shape; while adult human cardiomyocytes are large, highly organized, ~25% binucleated (Olivetti et al., 1996) with rod-like shape. In addition, hPSC-CMs possess sparse, disorganized and shorter sarcomeres (~1.6 μm), and few or no transverse tubules (T-tubules). Normal adult cardiomyocytes exhibit well-aligned, longer sarcomeres (~2.2 μm) characterized by the presence of Z discs, and I-, H-, A-, and M-bands.
3. Metabolically, hPSC-CMs are characterized by a relatively low number of mitochondria and a dependence on glycolysis as opposed to a predominantly fatty acid metabolism in adult cardiomyocytes (Yang et al., 2014a).
4. Functionally, hPSC-CMs display a force-generation capacity (0.22 ± 0.70 mN/mm^2^ −11.8 ± 4.5 mN/mm^2^) (Kita-Matsuo et al., 2009; Zhang et al., 2013) comparable to fetal cardiomyocytes (2nd trimester) (~0.4 mN/mm^2^) (Ribeiro et al., 2015) and much lower than adult (~51 mN/mm^2^) (Van Der Velden et al., 1998).
5. Electrophysiologically, hPSC-CMs show greater heterogeneity and immaturity in their electrical properties than adult cardiomyocytes including: (a) reduced electrical excitability; (b) decreased excitation–contraction coupling (ECC); (c) higher resting membrane potential (−20 to −60 mV vs. ~−90 mV); (d) low capacitance; (e) smaller upstroke (15–50 vs. 180–400 V/s) and conduction velocity (2.1–20 vs. 41–84 cm/s); and (f) presence of automaticity (spontaneous beating), which is found in early fetal cardiomyocytes and later specific to pacemaker cells.

**Table 1:** **Human pluripotent stem cell-derived cardiomyocytes (hPSC-CMs) vs. adult ventricular cardiomyocytes**.

**Criteria**			**hPSC-CM**	**Adult ventricular cardiomyocytes**	**References**
Structure	Shape		Round	Rod	Gerdes et al., 1992; Lundy et al., 2013
	Cell surface area		10212–14418 μm^2^	500–1294 μm^2^	Li et al., 1996; Lundy et al., 2013; Ribeiro et al., 2015
	Gene Expression		MYH7 < MYH6 TNNI3 < TNNI1	MYH7 > MYH6 TNNI3 > TNNI1	Xu et al., 2009
	Nuclei		Mononuclear	25% binucleation	Olivetti et al., 1996; Snir et al., 2003
	Sarcomere		~1.65 μm	~2.2 μm	Van Der Velden et al., 1998; Lundy et al., 2013
	T-tubules		Absent	Present	Brette and Orchard, 2003; Yang et al., 2014a
Energy and force	Mitochondria		Near nuclei, small fraction	Throughout cell; 20–40% of cell volume	Schaper et al., 1985; Gherghiceanu et al., 2011
	Energy		Glycolysis	ß-oxidation of fatty acid	Lopaschuk and Jaswal, 2010; Kim et al., 2013
	Contractile force		0.22 ± 0.70 to 11.8 ± 4.5 mN/mm^2^	51 ±8 mN/mm^2^	Van Der Velden et al., 1998; Kita-Matsuo et al., 2009; Zhang et al., 2013
	Proliferation		Early hPSC-CM: Yes Late hPSC-CM: No	Considered non-proliferative	Bergmann et al., 2009; Robertson et al., 2013
Calcium transients			Inefficient	Efficient	Itzhaki et al., 2011
Excitation-contraction coupling			Slow	Fast	Yang et al., 2014a
AP(action potential) properties	Upstroke velocity		15–50 V/s	180–400 V/s	Dangman et al., 1982; Drouin et al., 1995; He et al., 2003; Lundy et al., 2013
	Resting membrane potential		−20 to −60 mV	−90 mV	Drouin et al., 1995; Mummery et al., 2003; Lundy et al., 2013
	Conduction velocity		2.1–20 cm/s	41–84 cm/s	Nanthakumar et al., 2007; Caspi et al., 2009; Lee et al., 2012
	Capacitance		5–30 pF	150 pF	Drouin et al., 1995; Blazeski et al., 2012
	Automaticity		Spontaneous beating	Quiescent	Chen et al., 2009; Lundy et al., 2013
	mRNA level	Ca_*v*_1.2	Similar to adult cardiomyocyte	–	Satin et al., 2008
		Ca_*v*_ß1	20 fold lower than adult cardiomyocyte	–	Satin et al., 2008
		RyR2	~1000 fold lower than adult cardiomyocyte	–	Satin et al., 2008
	Ion channel density (pA/pF)	I_*Na*_	−20 to −330	~−50	Valdivia et al., 2005; Fatima et al., 2013; Ivashchenko et al., 2013
		I_*CaL*_	−2.2 to −11	−2.3 to ~−10	Magyar et al., 2000; Er et al., 2009; Fu et al., 2010; Otsuji et al., 2010
		I_*to*_	2.5–13.7	2.3–9.2	Beuckelmann et al., 1993; Wettwer et al., 1994; Ma et al., 2011; Cordeiro et al., 2013
		I_*Ks*_	0.3–0.7	0.18	Virag et al., 2001; Otsuji et al., 2010; Ma et al., 2011; Jonsson et al., 2012
		I_*Kr*_	0.4–0.8	0.6	Jost et al., 2009; Fu et al., 2011; Ma et al., 2011
		I_*K*1_	−0.6 to −3.4	~−12	Schram et al., 2003; Sartiani et al., 2007
		I_*NCX*_	3.6–7.9 (Ca^2+^ inward mode)	2.5–3	Weber et al., 2003; Fu et al., 2010

**Figure 1:**
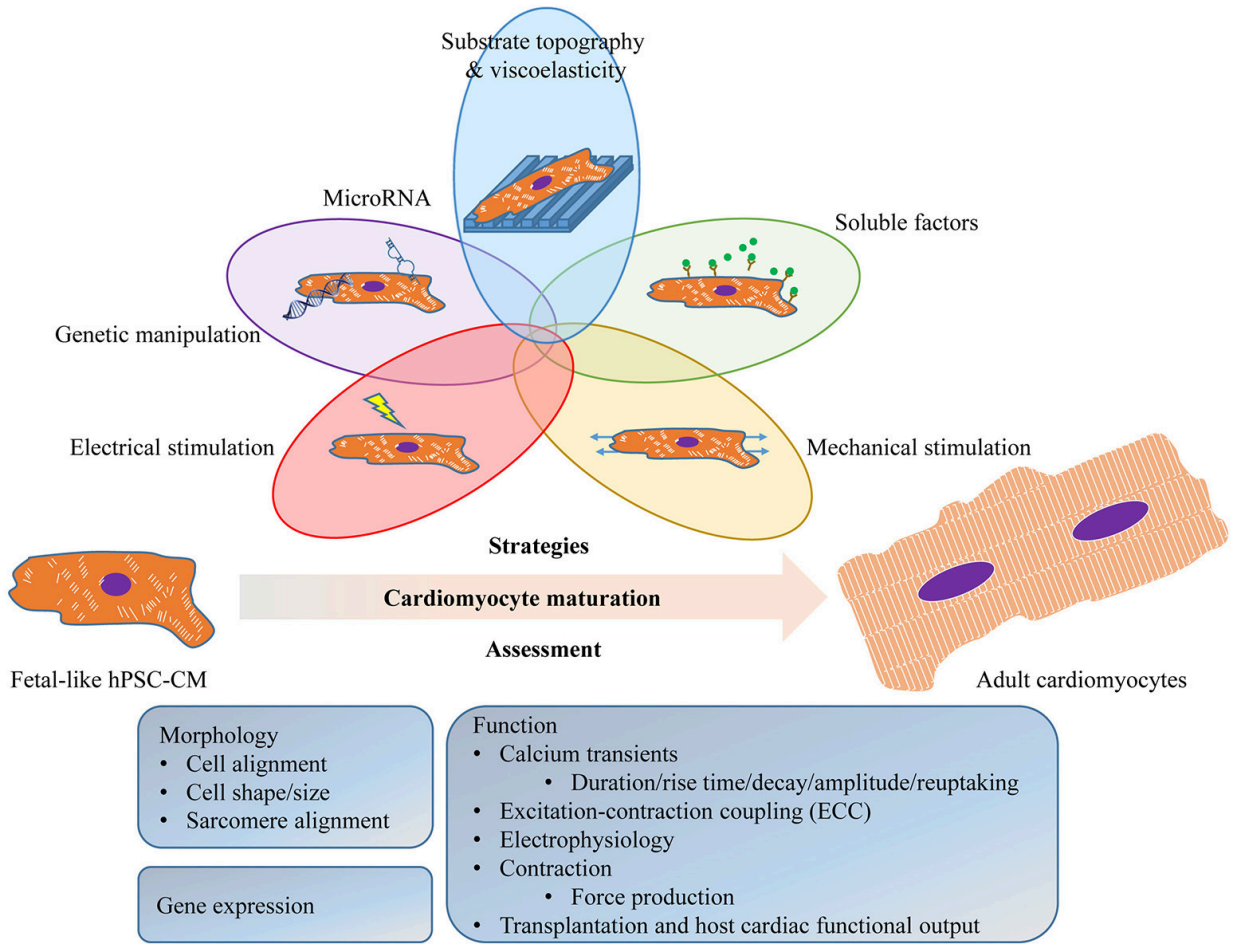
**Schematic diagram illustrating the strategies to promote and the assessment of the maturation of human pluripotent stem cell (hPSC)-derived cardiomyocytes (hPSC-CM)**. These approaches may be used individually or in any combination to promote hPSC-CM maturation. The assessment of the maturation should be physiologically relevant, including readout from morphology (cell alignment, cell shape/size, sarcomeres and T-tubules), gene expression (sarcomeric, ion channels and their regulators), and function (calcium handling, ECC, electrophysiology, contraction and transplantation).

These immature features may limit hPSC-CM application and highlight the need for the development of pro-maturation strategies to obtain human adult cardiomyocytes *in vitro*. Given the complexity of the cardiomyocyte structure and function, the term “maturation” represents multi-faceted properties used to evaluate their maturation state. However, the properties reported in different studies have often varied (Figure 1) making it difficult to draw a direct comparison.

## Strategies to induce hPSC-CM maturation

Cardiomyocytes undergo a series of structural changes and ultimately reach full maturity in the adult heart, which enables them to fulfill their functional role. This development process is long (years) and under complex regulation (Ahuja et al., 2007). hPSC-CMs could mature to adult-like size and morphology within 3 months post-transplantation into infarcted hearts of non-human primates (Chong et al., 2014). Long-term culture *in vitro* (80–120 days) has been suggested effective in improving the maturity of hPSC-CMs (Lundy et al., 2013). However, this is very time-consuming and cost prohibitive. More strategies to promote the maturity of hPSC-CMs include: genetic manipulation (e.g., adenovirus-mediated overexpressing of Kir2.1 Lieu et al., 2013), modulation of microRNAs (e.g., lentivirus-mediated overexpression of miR-1 Fu et al., 2011), delivery of biochemical factors, such as triiodothyronine (Yang et al., 2014b) and alpha-adrenergic agonist phenylephrine (Foldes et al., 2011), induction of cell alignment in 3D tissues (Zhu et al., 2014), and electrical and/or mechanical stimulation.

Of these, mechanical and electrical stimulation are major biophysical cues that play critical roles in cardiomyocyte growth and maturation during cardiac development and have been tested as maturation cues for hPSC-CM. To replicate electromechanical forces *in vitro*, hPSC-CMs are cultured in a biomimetic environment comparable to native cardiac microarchitecture and subjected to mechanical and/or electrical stimuli. The goal is to promote the maturity of hPSC-CMs while improving our understanding of the mechanisms responsible for the adaptive changes of cardiac tissue under physiological and pathological conditions.

### Mechanical stress

Mechanical force plays a critical role during development of cardiac structure and function (Zimmermann, 2013). It may thus be important to consider the presence of proper mechanical signaling or cues when designing a platform for the maturation of hPSC-CMs, regardless of whether it is in 2D or 3D. Mechanical stimulation on cells can be implemented by adjusting the substrate properties (stiffness/topography) and/or stretching. These have been suggested to be effective in improving the maturation properties of hPSC-CMs.

The effect of substrate rigidity on maturation can be demonstrated by plating spontaneously contracting hPSC-CMs on extracellular matrix (ECM) protein-coated tissue culture surfaces where the matrix composition can be altered to obtain physiological range of substrate stiffness. It's been shown that in a range of 4–80 kPa polyacrylamide hydrogels, the highest differentiation efficiency using hESCs was achieved at 50 kPa (Hazeltine et al., 2014), and that contractile output of cardiomyocytes increased in response to increased substrate stiffness (4.4–99.7 kPa) (Hazeltine et al., 2012). Two-dimensional substrates can also be micropatterned to improve hPSC-CM alignment and sarcoplasmic reticulum (SR) Ca^2+^ cycling (Rao et al., 2013; Salick et al., 2014), which suggest improved maturation. However, these two-dimensional structures lack important features of the natural 3D environment that affect the cell biology.

Stretch is the major method used to deliver mechanical stimuli to hPSC-CMs and generally done by applying external mechanical stress to hPSC-CM constructs in a static (achieved by increasing the stretch over time or directly to a fixed distance) or dynamic (mimicking the native cyclic mechanical stimulus on the cardiac muscle) fashion.

Early studies to test the effect of mechanical stress on immature cardiomyocytes were performed by seeding cells in collagen/Matrigel matrix, casting it in circular molds and, following tissue compaction, the engineered heart tissues (EHT) (Zimmermann et al., 2002) were subjected to uniaxial cyclic stretch (2 Hz, 10% elongation). After 1 week, EHTs displayed important hallmarks of mature myocardium: organized muscle bundles with aligned sarcomeres and positive force-frequency relationship (Endoh, 2004). Furthermore, these hEHTs show a positive inotropic response to extracellular Ca^2+^ and isoproterenol (Streckfuss-Bomeke et al., 2013).

In another study, hEHTs were generated by mixing single-cell hESC-CMs in a fibrin/Matrigel gel and casting into a 12 × 3 × 3 mm agarose mold in which two elastic silicone posts were inserted from above (Schaaf et al., 2011). Upon compaction, the cardiac construct strip anchored to the posts was subjected to static strain and displayed improved cell alignment and sarcomeric organization compared with age-matched EBs, and expressed connexin-43 but not in intercalated disks. Transcription levels of β-MHC increased significantly over time in hEHTs but not in EBs. The hEHTs demonstrated contractions 5–10 days after casting, reached regular (mean 0.5 Hz) and strong (mean 100 mN) contractions for up to 8 weeks. The constructs exhibited positive chronotropic and inotropic response to increasing concentrations of extracellular Ca^2+^ (Schaaf et al., 2011).

Cardiac constructs were also generated by casting collagen-based hPSC-CMs gels in a 20 mm × 3 mm channel, in which the ends of the construct were anchored into nylon mesh tabs attached to a deformable silicon floor of the well (tissue train, Flexcell). Upon cell remodeling and gel contraction, the cardiac constructs were held by the nylon tabs under static tension or subjected to controlled cyclic stress (1 Hz, 5% elongation) (Tulloch et al., 2011). After 4 days, there was improvement in cell alignment and striations within the constructs. Cyclic stretch also upregulated transcripts of β-MHC, cTnT, ANP, BNP, CACNA1C, RYR2, and SERCA2 (Tulloch et al., 2011). Functionally, cardiac constructs subjected to 3 weeks of static strain have increased their active force in response to increased resting length (Tulloch et al., 2011), analogous to Frank-Starling curves (an increase in force with increased preload known as length-dependent activation) (Glower et al., 1985).

Mihic et al. (2014) used cyclic mechanical stretch to enhance the viability and functional maturation of hPSC-CM tissue constructs prior to implantation into the damaged myocardium. The constructs were generated by seeding hESC-CMs in a 30 × 10 × 7 mm gelatin sponges. After 2 days of compaction, the cardiac constructs were subjected to 3 days of uniaxial cyclic stretch (1.25 Hz, 12% elongation). Compared to unstretched controls, cyclically stretched cardiac constructs exhibited increased number of cells, cell size and elongation, increased expression of connexin-43, and upregulated mRNA expression of MYH7, CACNA1C, HCN4, KCNH2, SCN5A, and KCNJ2. Functionally, the cyclically stretched cardiac constructs were demonstrated faster contraction rates with shorter calcium cycle duration.

Zhang et al. (2013) used a platform to promote hESC-CMs alignment within cardiac patch via locally controlling the direction of passive tension. hESC-CMs (48–90% purity) were cultured for 2 weeks in a mixture of fibrin and Matrigel in 7 × 7 mm^2^ polydimethylsiloxane (PDMS) molds with staggered hexagonal posts (1.2 mm long) to generate a cardiac patches with elliptical pores formed around the posts upon tissue compaction. The resultant hESC-CMs in the 3D patches exhibited a maximal conduction velocity of 25.1 cm/s, and longer sarcomeres (2.09 ± 0.02 vs. 1.77 ± 0.01 μm), and enhanced expression of genes involved in cardiac contractile function, including cTnT, αMHC, CASQ2 and SERCA2 when compared to age and purity matched hESC-CMs cultured in monolayers (Zhang et al., 2013). Moreover, maximum contractile forces and active stresses of cardiac patches were 3.0 ± 1.1 mN and 11.8 ± 4.5 mN/mm^2^, respectively, and the patches were shown to generate Frank-Starling curves with respect to both active and passive force as well as positive inotropic response to isoproterenol (Zhang et al., 2013). These author's findings highlight the superiority of 3D vs. 2D culture models. However, no improvements in the electrophysiological properties were reported.

These studies have established the significance of mechanical stimuli as a maturation cue for hPSC-CMs. However, it should be noted that the contractile forces measured from the aforementioned EHTs were related to the biomaterial composition (e.g., collagen vs. fibrin). Such material variability may affect the hPSC-CM phenotype, which consequently cause the variation of functional readout including contractile force. Furthermore, other variables in these mechanical stimulation regimes, such as the cell culture condition and duration of stimulation, makes it difficult to determine an optimal mechanical stress protocol for generating mature cardiac tissues.

### Electrical stimulation

Cardiomyocytes are rhythmically and synchronously contracting in response to electrical signals. This process of converting electrical signals into contraction (commonly known as excitation-contraction coupling or ECC) requires the coordinated activity of several ion channels (Liu et al., 2016). The developmental changes in these ion channels are under complex regulation and accompany changes in electrical properties of cardiomyocytes across the fetal and postnatal stages, with a specific electrophysiological “signature” in mature adult cardiomyocytes. hPSC-CMs have been shown to be electrophysiologically immature. Studies recapitulating *in vitro* the electrical activity cardiomyocytes are exposed to *in vivo* have demonstrated that electrical stimulation promotes aspects of hPSC-CM maturation.

We have devised a platform called “biowire,” to mature hPSC-CMs by combining 3D culture and electrical stimulation (Nunes et al., 2013; Sun and Nunes, 2016). Biowires were generated by culturing hPSC-CMs in collagen hydrogels around a surgical suture to form cardiac tissues of ~600 μm in diameter (Nunes et al., 2013). Biowires were subjected to 7 days of electrical field stimulation (3 V/cm, 1 ms pulse, starting at 1 Hz with step-wise increases to 3 or 6 Hz). At the endpoint, hPSC-CMs exhibited properties compatible with cardiomyocyte maturation, such as improved cell and myofibril alignment, improved sarcomeric banding, larger cardiomyocyte area and lower proliferation rates, compared with age-matched EBs. Automaticity was significantly higher in EB-derived cardiomyocytes compared to control biowires, which was comparable to that in biowires subjected to the 6-Hz regimen. Electrical stimulation also significantly increased the conduction velocity of biowires from ~11.5 to 18.5 cm/s. Biowires exposed to electrical stimulation also showed increased Ca^2+^ transient amplitudes vs. unstimulated controls. hPSC-CMs in biowires exhibited improved hERG current and inward rectifier current (*I*_k1_) densities, which were further enhanced by electrical stimulation. This study revealed for the first time that these changes were dependent on the electrical stimulation rate as evidenced by greater extent of maturation obtained in the biowires exposed to the 6 Hz stimulation ramp-up regimen (vs 3 Hz) (Nunes et al., 2013). However, given the presence of the silk suture the force of contraction generated by the hPSC-CMs could not be measured. The use of a biodegradable suture may make this possible in the future.

Others have shown that hESC-CMs subjected to 2-week-long electrical conditioning (2.5 V/cm, 1 Hz, 5 ms pulse) exhibited lower spontaneous activity, hyperpolarized resting potential, increased intracellular Ca^2+^ transients, structured organization of myofilaments, and an upregulation of Kir2.1, CSQ2, junctin, triadin, SERCA, Cav3, Amp2, MHC, and MLC genes (Lieu et al., 2013). In another study, beating EBs seeded on gelatin-coated plates and subjected to 4-day-long electrical stimulation (6.6 V/cm, 1 Hz, 2 ms pulse) exhibited cell elongation, increased action potential duration, increased Ca^2+^ transients and increased expression of cardiac-specific gene including HCN1, MLC2V, SCN5A, SERCA, Kv4.3, and GATA4 (Chan et al., 2013).

In a recent study, EBs differentiated from hPSCs were subjected to electrical conditioning (5 V/cm, 0.5, 1 and 2 Hz, 2 ms pulse) continuously for 7 days (Eng et al., 2016). Such electrical stimulation enhanced connexin expression and sarcomeric structure. Cardiomyocytes adapted their autonomous beating rate to the frequency at which they were stimulated, an effect mediated by the emergence of a rapidly depolarizing cell type, and the expression of hERG. The resultant cardiomyocytes were robust and could maintain the adapted beating rates for up to 2 weeks after the cessation of electrical stimulation (Eng et al., 2016).

While electrical stimulation has consistently improved the maturation of hPSC-CMs, one possible drawback of utilizing electrical stimulation is the limited scalability. This may not be of concern for its utilization in drug screening platforms but may hinder its application in cell maturation for regenerative medicine applications.

### Combined mechanical and electrical stimulation

Efforts have also been made to examine the effect of combining mechanical and electrical stimulation, sequentially or concurrently, to hPSC-CM constructs. Hirt et al. (2014) generated spontaneously beating fibrin/Matrigel-based hPSC-CM constructs with static stretch and subjected them to electrical field stimulation (2 V/cm, 4 ms pulse, 2 Hz for 1 week and 1.5 Hz thereafter) for at least 10 days. This increased cell alignment, sarcomere organization, Ca^2+^-response curves, force generation and inotropic response to β-adrenergic stimulation while decreasing automaticity. (Hirt et al., 2014).

In another study, hPSC-CMs were embedded into a collagen-based scaffold and then subjected to static stress for 2 or 1 week of static stress and 1 week of combined static stress and electrical pacing (5 V/cm, 2 Hz, 5 ms pulse) (Ruan et al., 2016). Compared to no stress/no pacing controls, 2-week static stress conditioning promoted cell alignment, passive stiffness, cardiac hypertrophy, and increased contractility of hPSC-CM constructs (0.63 ± 0.10 mN/mm^2^ vs. 0.055 ± 0.009 mN/mm^2^). The contractility of the constructs could be further increased by combining stretch with 1-week electrical stimulation (1.34 ± 0.19 mN/mm^2^). Combined static stress and electrical stimulation enhanced expression of SR-related proteins (RYR2 and SERCA2) (Ruan et al., 2016).

## Conclusions and future directions

The efforts to mimic native biophysical stimulation to mature hPSC-CMs have led to a number of effective strategies to mature hPSC-CMs and advance our understanding of how these cues affect cardiomyocyte structure and function. However, the properties assessed often varied between studies making it difficult to draw a direct comparison between the different strategies. This is accentuated by the lack of uniformity in cardiomyocyte maturation in artificial, *in vitro* settings where electrical stimulation seems to have a stronger impact on electrical properties while mechanical stimulation improves structural components and force generation with smaller impact on electrical properties. This argues for a homogeneity in the parameters utilized as functional readouts (electrophysiology, calcium dynamics, force of contraction and ultrastructure).

Although progress has been made, an adult-like phenotype *in vitro* has yet to be reported. This can have multiple limitations regarding application. First, the maturation status of hPSC-CMs should be staged and documented depending on the potential application sceneries. For example, for myocardial infarction (MI) therapy, less mature cardiomyocytes might adapt better for transplantation into the infarcted myocardium (Reinecke et al., 1999). However, the best-defined maturation stage of hPSC-CMs for transplantation into MI remains to be determined.

Second, the hPSC-CMs obtained from existing cardiac differentiation protocols are a mixed population of ventricular-, atrial-, and nodal-like cells. Such heterogeneity represents a limitation for certain applications, e.g., transplantation of high purity of ventricular cardiomyocytes to potentially avoid tachyarrhythmias caused by spontaneously firing (nodal-like) cells; and high throughput (HTS) drug testing platforms for cardiac drug responses.

Third, the significance of the *in vivo* environment for the maturation of cardiomyocytes should be noted. Immature hPSC-CMs differentiated *in vitro* could mature to adult size and morphology after transplantation into the infarcted hearts of non-human primates (Chong et al., 2014).

Proper cardiac development and function requires other cell types, such as fibroblasts, endothelial, and smooth muscle cells that may have an impact in cardiomyocyte maturation. While there is still controversy regarding whether non-cardiomyocytes may promote hPSC-CM maturation via secretion of undefined factors (Kim et al., 2010; Lundy et al., 2013), a full understanding of these interactions may help to uncover unknown cues, which could then be used to promote hPSC-CM maturation in the absence of a specific cell type.

hPSC-CMs have shown great promises in various applications including cardiac development, regenerative medicine, disease modeling, and drug testing/screening/discovery. The generation of a large number of mature hPSC-CMs is essential to achieve these goals. Importantly, these approaches are not mutually exclusive (Figure 1) and there's been a trend to combine the existing strategies to obtain more effective maturation. The combination of mechanical and electrical stimulation has shown possible synergistic effects with a 2-fold increase in contractility (Ruan et al., 2016). This trend should lead to exciting discoveries regarding hPSC-CM maturation and possibly the achievement of adult-like cardiomyocytes *in vitro* for the first time.

## Author contributions

XS and SN conceived and wrote the manuscript.

## Funding

This work was supported by a grant-in-aid from the Heart and Stroke Foundation of Canada (G-14-0006265), operating grants from the Canadian Institutes of Health Research (137352 and 143066) and a J.P. Bickell foundation grant (1013821) to SN.

### Conflict of interest statement

The authors declare that the research was conducted in the absence of any commercial or financial relationships that could be construed as a potential conflict of interest.
